# Extending Biopython to combine multiple sequence alignments with the same reference into a Multiple Sequence Alignment.

**DOI:** 10.17912/micropub.biology.001966

**Published:** 2026-02-18

**Authors:** Cassia Bastress, Michiel de Hoon, Manuel Lera-Ramirez, Jürg Bähler

**Affiliations:** 1 Faculty of Science, McGill University, Montréal, Canada; 2 RIKEN Center for Integrative Medical Sciences, Yokohama, Japan; 3 University College London, London, United Kingdom

## Abstract

Pairwise alignments (PWAs) are commonly used to compare sequences to a reference. Existing alignment tools provide algorithms to align multiple sequences to a single reference and to merge two sets of aligned sequences; but not to combine individually aligned PWAs with a common reference into a single MSA which preserves their original alignment structure. This is required for certain workflows. One example is aligning multiple sequencing traces with a circular plasmid sequence for validation. Some alignment tools that take into account the circularity of the plasmid sequence return a PWA per sequencing trace. For visualization, all PWAs have to be combined into a single MSA. For this purpose, we developed an algorithm that combines alignments sharing the same reference into an MSA, and implemented it as a classmethod in Biopython’s Alignment class.

**Figure 1. Example workflow for combining Sanger sequencing traces into a multiple sequence alignment:**
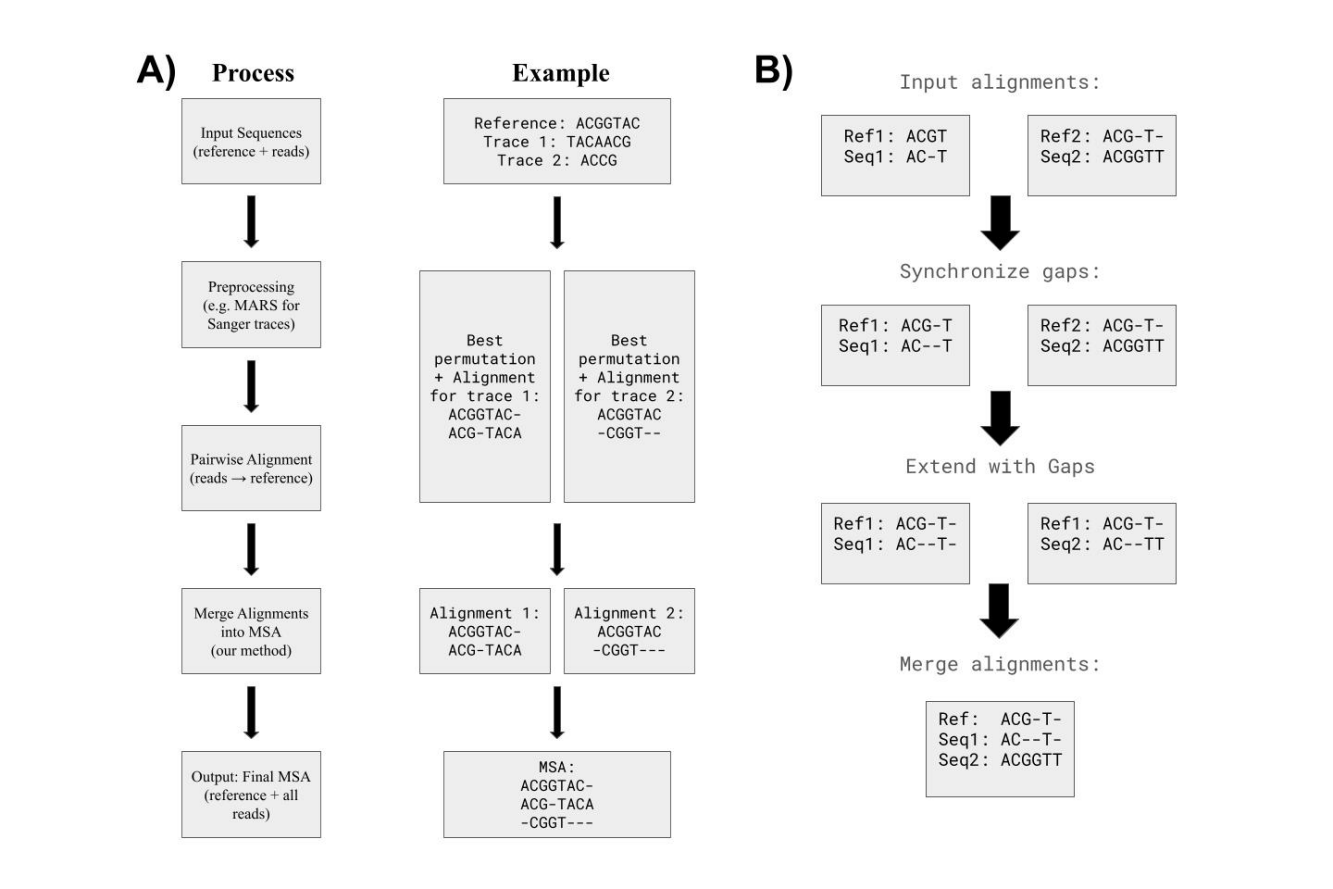
(A) Example workflow for generating a multiple sequence alignment (MSA) from Sanger sequencing traces that may span the origin of a circular reference. The workflow includes preprocessing of traces (e.g., using MARS to permute sequences), pairwise alignment of each trace to the reference, selection of the best pairwise alignment (PWA), and finally merging the PWAs into an MSA using from_pairwise_alignments. An example with small sequences is shown for clarity.&nbsp; (B) Example illustrating how the method from_pairwise_alignments combines pairwise alignments into a multiple sequence alignment (MSA). The figure shows an example input of two pairwise alignments, how gaps are synchronized across them, how each alignment is extended with gaps to achieve consistent lengths, and finally how the synchronized alignments are merged into an MSA. For a detailed step-by-step description of the algorithm, see Extended Data.

## Description

Sequence alignment enables the comparison of DNA, RNA, or protein sequences to identify regions of similarity, which can provide insight into structural features or evolutionary relationships. Sequence alignment can also be used to validate that engineered DNA constructs produced in the laboratory match their intended designs by aligning sequencing reads to DNA sequences designed *in silico* . Design validation involves aligning each read individually to the reference, and combining these results into a consolidated view that shows all reads against the reference simultaneously. This type of alignment only minimizes the differences of reads with respect to the reference, and not between reads themselves.

Widely used tools such as Clustal Omega (Sievers et al., 2011), MUSCLE (Edgar, 2004), MAFFT (Katoh et al., 2002), and FAMSA2 (Gudyś et al., 2025) provide algorithms for both pairwise and multiple sequence alignment. MAFFT in particular supports aligning multiple sequences to a reference, but does not provide a way to directly merge a collection of previously generated alignments into a single MSA. FAMSA2 can combine several MSAs into a single MSA, but in doing so it also aligns non-reference sequences among them. This is the desired outcome when using alignments for phylogenetic analysis, but not for validating genetic constructs aligning them with sequencing data where we want to retain the pairwise comparison of the reference and the queries. This is especially important for Sanger sequencing, where a lot of noise is found at the edges of traces.&nbsp;&nbsp;

These tools also have limitations when aligning plasmids to sequencing traces. Traces may match the forward or reverse strand of the plasmid, and span the origin. Alignment tools do not take into account circularity of sequences, so if the reference sequence is circular (the plasmid), it may need to be permuted before alignment with traces. For this task, we use a simple pipeline:&nbsp;

This approach produces a set of PWAs (one per sequencing trace), each mapped to the same reference sequence. To visually analyze the results, we needed a way to combine all the PWAs into a single MSA while maintaining the original alignment structure. Fig.1A shows how the new method addresses this final step by providing a robust way to merge a set of alignments into an MSA.

We implemented this functionality as a class method in Biopython (Cock et al., 2009). Biopython is a collection of open-source Python tools for biological computation which provides classes to represent both PWAs and MSAs. It has not until now included a method to combine a set of alignments into a single MSA.

## Methods

We extended the Biopython library by adding a class method, from_alignments_with_same_reference, to the Alignment class. This method takes as input a list or tuple of Alignment objects and returns a single MSA represented as an Alignment object. All input alignments must share the same reference sequence (ignoring gaps). This functionality allows users to combine individual alignments to a reference into a single MSA.

The algorithm works as follows (see Fig. 1B ):

For a detailed step-by-step description of the algorithm, see Extended Data.

Algorithm Complexity

Let S denote the total number of sequences with the shared reference counted only once, and let N be the alignment length. The dominant cost arises from the main coordinate-synchronization loop, which in the worst case, has O(SN) iterations. Each iteration performs O(S) work to update and synchronize coordinates, yielding an overall worst-case complexity of O(S ^2^ N). Memory usage is O(SN) as the algorithm explicitly stores the full coordinate representation of the final MSA, which has S sequences and N alignment columns. In comparison, the general complexity for the default progressive method (FFT-NS-2) from MAFFT is O(S ^2^ N) + O(N ^2^ S) (Katoh & Toh, 2008).

## Data Availability

Description: Document describing the principle of the algorithm with examples.. Resource Type: Text. DOI: https://doi.org/10.22002/qnbhp-4jc30 Description: Slides complementing the Algorithm Narration.. Resource Type: Image. DOI: https://doi.org/10.22002/vpn8n-fb612

## References

[R1] Ayad Lorraine A. K., Pissis Solon P. (2017). MARS: improving multiple circular sequence alignment using refined sequences. BMC Genomics.

[R2] Cock Peter J. A., Antao Tiago, Chang Jeffrey T., Chapman Brad A., Cox Cymon J., Dalke Andrew, Friedberg Iddo, Hamelryck Thomas, Kauff Frank, Wilczynski Bartek, de Hoon Michiel J. L. (2009). Biopython: freely available Python tools for computational molecular biology and bioinformatics. Bioinformatics.

[R3] Edgar R. C. (2004). MUSCLE: multiple sequence alignment with high accuracy and high throughput. Nucleic Acids Research.

[R4] Gudyś Adam, Zielezinski Andrzej, Notredame Cedric, Deorowicz Sebastian (2025). FAMSA2 enables accurate multiple sequence alignment at protein-universe scale.

[R5] Katoh K. (2002). MAFFT: a novel method for rapid multiple sequence alignment based on fast Fourier transform. Nucleic Acids Research.

[R6] Katoh K., Toh H. (2008). Recent developments in the MAFFT multiple sequence alignment program. Briefings in Bioinformatics.

[R7] Sievers Fabian, Wilm Andreas, Dineen David, Gibson Toby J, Karplus Kevin, Li Weizhong, Lopez Rodrigo, McWilliam Hamish, Remmert Michael, Söding Johannes, Thompson Julie D, Higgins Desmond G (2011). Fast, scalable generation of high‐quality protein multiple sequence alignments using Clustal Omega. Molecular Systems Biology.

